# Metalliferous Biosignatures for Deep Subsurface Microbial Activity

**DOI:** 10.1007/s11084-015-9466-x

**Published:** 2015-09-16

**Authors:** John Parnell, Connor Brolly, Sam Spinks, Stephen Bowden

**Affiliations:** Department of Geology & Petroleum Geology, University of Aberdeen, Aberdeen, UK; CSIRO Mineral Resources Flagship, Australian Resources Research Centre, Perth, Australia

**Keywords:** Deep subsurface, Deep biosphere, Reduction spheroid, Iron-reducing bacteria, Metalliferous biosignature, Selenium, Raman spectroscopy

## Abstract

The interaction of microbes and metals is widely assumed to have occurred in surface or very shallow subsurface environments. However new evidence suggests that much microbial activity occurs in the deep subsurface. Fluvial, lacustrine and aeolian ‘red beds’ contain widespread centimetre-scale reduction spheroids in which a pale reduced spheroid in otherwise red rocks contains a metalliferous core. Most of the reduction of Fe (III) in sediments is caused by Fe (III) reducing bacteria. They have the potential to reduce a range of metals and metalloids, including V, Cu, Mo, U and Se, by substituting them for Fe (III) as electron acceptors, which are all elements common in reduction spheroids. The spheroidal morphology indicates that they were formed at depth, after compaction, which is consistent with a microbial formation. Given that the consequences of Fe (III) reduction have a visual expression, they are potential biosignatures during exploration of the terrestrial and extraterrestrial geological record. There is debate about the energy available from Fe (III) reduction on Mars, but the abundance of iron in Martian soils makes it one of the most valuable prospects for life there. Entrapment of the microbes themselves as fossils is possible, but a more realistic target during the exploration of Mars would be the colour contrasts reflecting selective reduction or oxidation. This can be achieved by analysing quartz grains across a reduction spheroid using Raman spectroscopy, which demonstrates its suitability for life detection in subsurface environments. Microbial action is the most suitable explanation for the formation of reduction spheroids and may act as metalliferous biosignatures for deep subsurface microbial activity.

## Introduction

The concentration and deposition of metals by microbial activity has given rise to a wide range of ore deposits (Fallick et al. 2001; Southam and Saunders 2005; Jamieson et al. 2013). In most cases, the interaction of microbes and metals is assumed to have occurred in surface or very shallow subsurface environments. However, it has become apparent that much microbial activity occurs in the deep subsurface below continents so that at about 2 km depth, pore fluids typically contains 10^4^ cells/ml (Whitman et al. 1998; McMahon and Parnell 2014). Thus, the microbial concentration of metals is likely to extend to the subsurface, and offers the potential to act as a signature for subsurface microbial activity in the geological record.

**Fig. 1:**
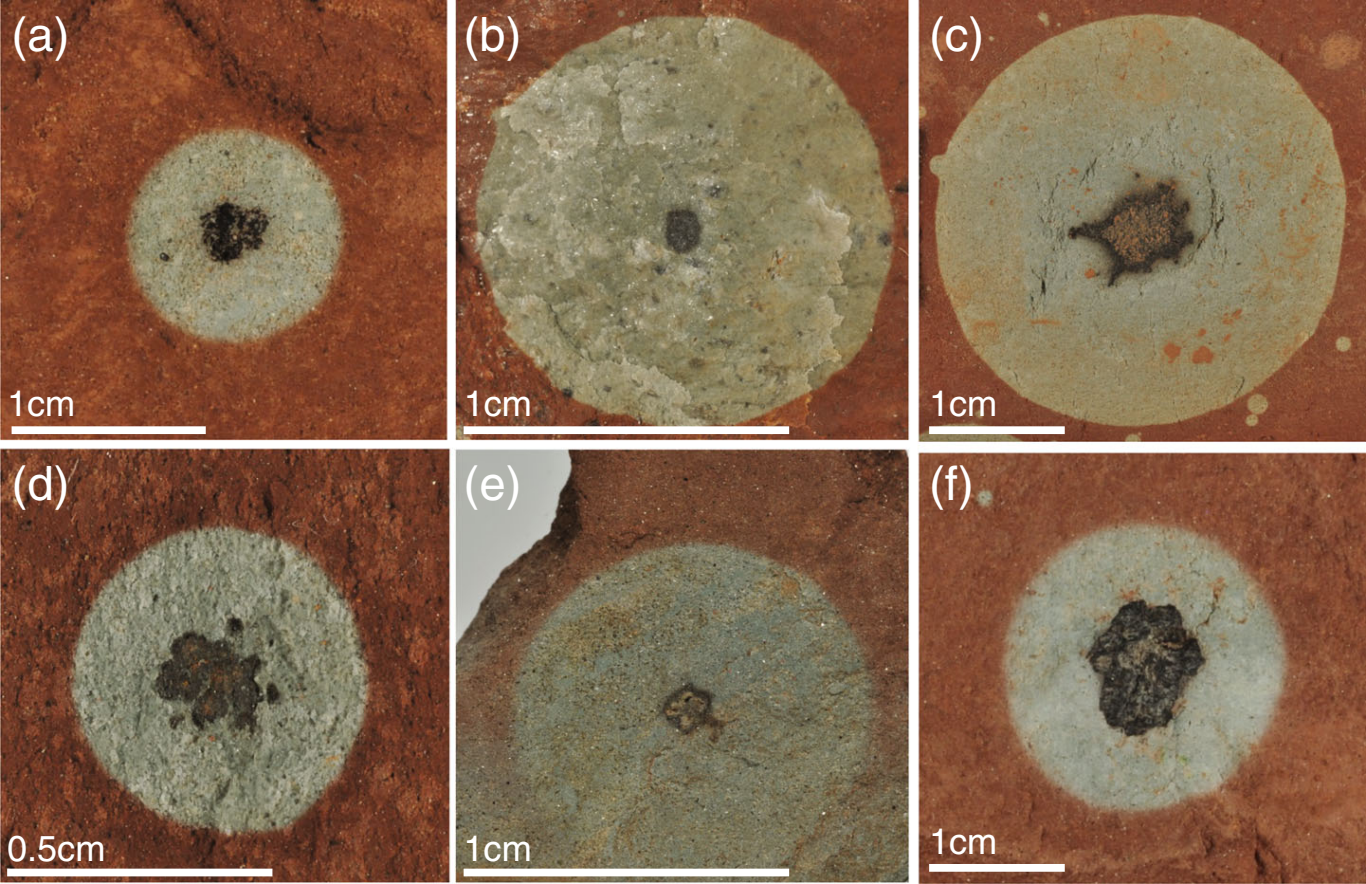
Examples of cm-scale Triassic reduction spheroids, with dark metal-rich cores. Reduced halos display almost complete sphericity. **a & f** Sidmouth, Devon. **b** Fauld mine, Staffordshire. **c** Okehampton, Devon. **d** Bantycock mine, Nottingham

One type of metalliferous deposit that is distinctly subsurface is in continental strata, the so-called ‘red beds’ deposits in fluvial, lacustrine and aeolian environments in which red colour is conferred from iron oxide coatings around the sand grains. In these rocks, metals are concentrated at redox boundaries between oxidised red and reduced pale-coloured rocks. The redox-sensitive metals and metalloids concentrated at these boundaries especially include V, Cu, Mo, U and Se, but a range of other elements, including tellurium, rare earth elements and platinum group elements are also enriched at such boundaries. On an aquifer scale, the boundaries form ‘roll-front’ deposits, where oxidizing groundwaters penetrate sandstones containing reductants (detrital organic matter, oil, hydrogen sulfide). These deposits can be large enough to mine for metals (e.g., Northrop and Goldhaber 1990; Min et al. 2005). On a much smaller scale, red beds contain widespread centimetre-scale reduction spheroids in which a pale reduced spheroid in otherwise red rocks contains a metalliferous core (e.g., Hofmann 1990), as shown in Fig. 1.

At both scales, a microbial origin for the metal deposition has been inferred (Northrop and Goldhaber 1990; Hofmann 1991). As the host rocks are usually fully compacted, metal deposition and related microbial activity took place in the deep subsurface.

## The Role of Iron-Reducing Bacteria

The defining characteristic of reduction spheroids and other reduction features in otherwise red rocks is the conversion of Fe (III) to Fe (II) mineralogy. Most of the reduction of Fe (III) in sediments is caused by Fe (III)-reducing bacteria (Lovley 1997). Where this bacterial activity is extensive, Fe (III) is reduced to Fe (II), and leached away, removing the red colouration associated with oxidised iron (Lovley 1997), and develops mottling of reduced and oxidised sediment in the subsurface. This mottling is observed in the geological record, which is thus reasoned to reflect microbial activity (Lovley et al. 1990). The widespread *Geobacter metallireducens* can seek out new Fe (III) oxide surfaces through chemotactic development of flagella (Childers et al. 2002), and hence colonies can exploit a volume of sediment and efficiently reduce it. The deposition of metals by reduction in the subsurface is achieved particularly through the activity of the Fe (III)-reducing bacteria. They have the potential to reduce a range of other metals and metalloids, including V, Cu, Mo, U and Se, by substituting them for Fe (III) as electron acceptors (Coates et al. 1996; Lovley 1997). These are all elements concentrated in red bed deposits, consistent with their purported microbial origin.

The source of the metals may be the iron oxide coatings that the bacteria strip off the sand grains. Iron oxides are major sinks for metals, liberated as labile minerals are altered during burial (Zielinski et al. 1986; Rose and Bianchi-Mosquera 1993). It is possible that the metal-rich iron oxide coatings were formed with the aid of other bacteria coating the sand grains, as most subsurface bacteria reside on grain surfaces (McMahon and Parnell 2014). Once sand grains are coated with iron oxide, the coatings may engender greater bacterial loading (Lukasik et al. 1999; Bolster et al. 2001). Biofilms on sand grains in turn enhance the uptake of metals (Diels et al. 2003), so the inorganic and organic components of the grain coatings are mutually reinforcing in sequestration of metal. Experimental evidence confirms that Fe (III) reducers are effective in dissolution of trace metals in the iron oxides (Zachara et al. 2001). The concentration of metals on grain coating iron oxides is comparable to the uptake of metals on Fe (III)-mineralised iron plaque around plant roots (Hansel et al. 2001), which is similarly susceptible to removal by Fe (III) reducing bacteria (Weiss et al. 2004).

Fe (III) reducing bacteria are ideally suited for subsurface growth. Many species are thermophilic, and so occur in the deep subsurface, up to several kilometres depth (Boone et al. 1995; Liu et al. 1997; Kieft et al. 1999; Holden and Feinberg 2005; Zhang et al. 2009). Experimental reduction of metals by Fe (III) reducers at 100 °C has implicated a microbial origin for uranium ore deposits (Kashefi and Lovley 2000). Fe (III) reduction is the predominant redox process in microfracture systems that are ubiquitous in the subsurface (Kinner et al. 2005). In summary, the reduction morphology, mineral and metal assemblage, and deep subsurface habitat of Fe (III) reducing bacteria all implicate their role in the formation of red bed-hosted metal deposits. Confirmatory evidence for a microbial origin for the deposition of metallic phases comes from the sulfur isotopic compositions of sulfides in the deposits. Pyrite (iron sulfide) from roll front and other red bed hosted deposits (Northrop and Goldhaber 1990; Spinks et al. 2015) and reduction spheroids (Spinks et al. 2010) has isotopic compositions much lighter than precursor sulfate, i.e., a degree of isotopic fractionation indicative of microbial sulfate reduction (Machel 2001).

## The Origin of Reduction Spheroids

**Fig. 2:**
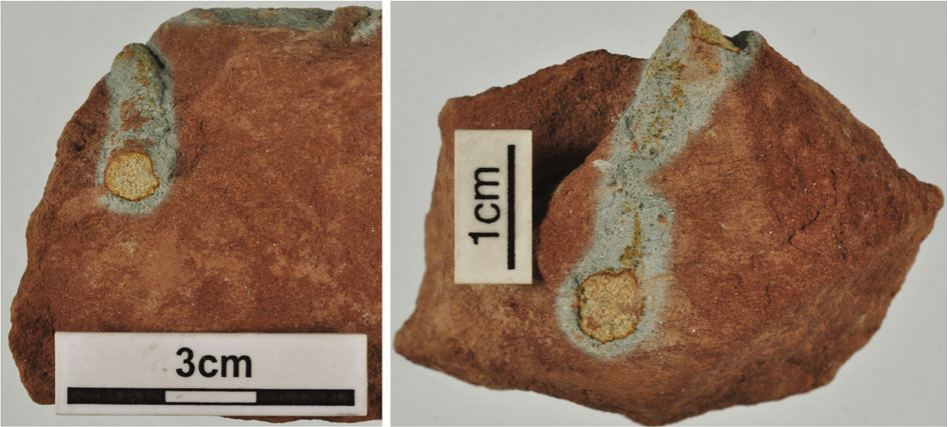
Reduction around plant roots in Triassic siltstone. Reduction follows the morphology of reductant and therefore is elongate

Reduction spheroids are very widely distributed in red beds, and are the most consistently encountered morphology of reduced sediment. The concentration of metals within them has been described in several case studies (e.g., Harrison 1975; Dyck and McCorkell 1983; Parnell 1988; Spinks et al. 2014). The metalliferous mineral phases are typically oxides, silicates or native elements (Harrison 1975; Hofmann 1990), rather than the sulfides that host metals in most other environments. Pyrite, which is ubiquitous in roll front deposits, is very rare in reduction spheroids. Selenides are encountered as widely as sulfides (Hofmann 1990), despite the much lower crustal abundance of selenium compared to sulfur. The spheroids have been attributed a microbial origin by Hofmann (1990, 1991) and by Spinks et al. (2010). The morphology of reduction spheroids is entirely consistent with a microbial origin. The distinctive features exhibited by spheroids are a halo of reduced iron oxide, and in some cases concentric bands enriched in metals. Bacterial colonies, including Fe (III) reducing bacteria, in plated growth media exhibit comparable haloes (Coates et al. 1998; Pham et al. 2003; Maki et al. 2012), and multiple concentric bands of growth and inhibition related to diffusion of ions from a central metallic source (Feeney et al. 1957; Matsushita et al. 1998; Lacasta et al. 1999). Reduction is also feasible through inorganic processes, but other characteristics of reduction spheroids favour a microbial origin. The spheroids developed post-compaction, not during early burial. Where reduction occurs around detrital organic matter, such as plant debris, it does not exhibit the spheroidal form, and is more likely to be lensoid, reflecting development at an early stage (as shown in Fig. 2).

The spheroidal form also indicates formation in a static pore fluid, rather than from a moving pore fluid which would lead to an asymmetric form. The spheroids are formed in host sediments with a wide range of grain sizes from mudrocks to conglomerates. This range represents a range of permeabilities and potential as aquifers, which again indicates that their formation is not dependent upon fluid flow. Instead, the spheroids must reflect a trigger for the reduction process, which in the absence of any visible nucleus is likely to be the bacteria that are both abundant and adapted to reduce metals.

Reduction haloes and associated metal concentrations are predominantly developed in continental strata, where the rocks are typically red. However, they also occur in deep marine sediments (Thomson et al. 1989, 1994), and in crystalline basement rocks (Hofmann 1990). These occurrences further suggests that it is the nature of the reductant, inferred to be the ubiquitous Fe (III) reducing bacteria, rather than an aspect of the environment, that is responsible for their formation.

**Fig. 3:**
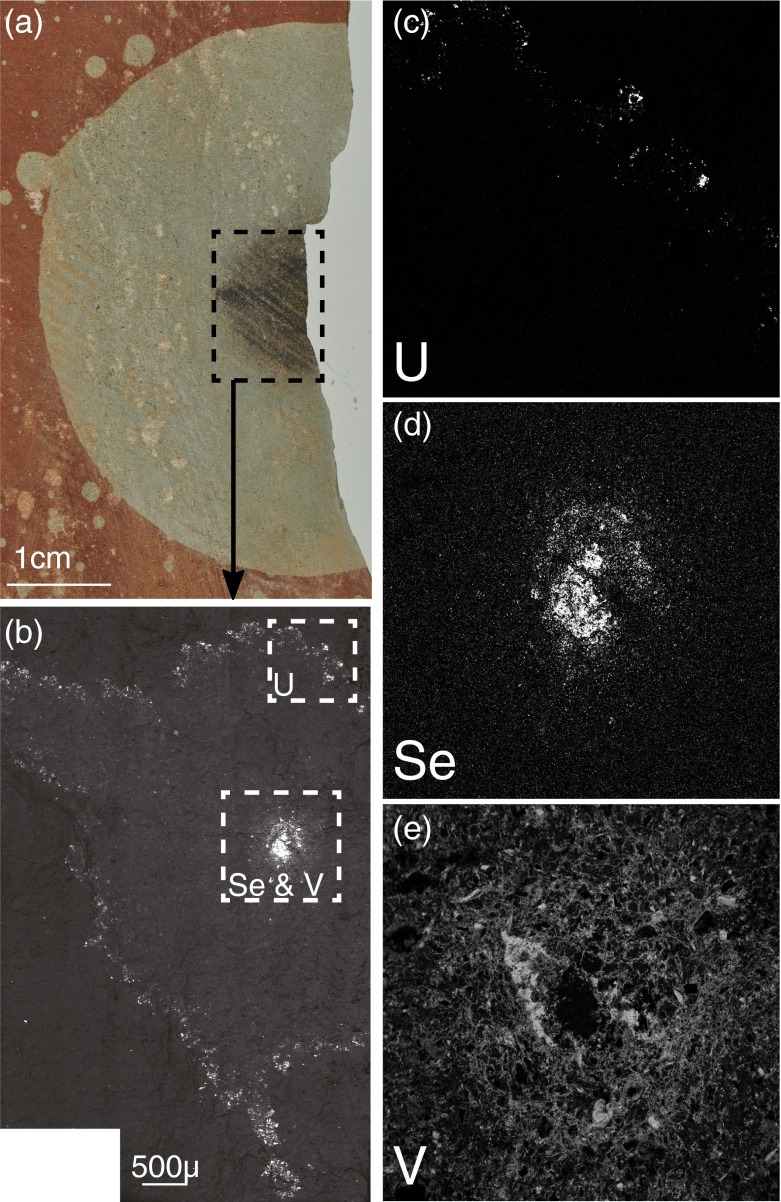
**a** Permian reduction spheroid from Exmouth, Devon with a dark metalliferous core. **b** Scanning electron microscope (SEM) photomontage of the metal rich core using back-scatter electron analysis. Bright areas represent elements with high atomic numbers. **c** Uranium (U) element map from outer ring of core. **d** Selenium (Se) element map from central core. **e** Vanadium (V) element map from central core. *Bright areas* represent higher concentrations of elements, and *dark areas* represent low concentrations. Element maps show marked concentrations of each element

Where they are associated, Fe (III) reducing bacteria may competitively exclude sulfate reducers (Chapelle and Lovley 1992), which may explain why sulfides rarely occur in reduction spheroids. However, Fe (III) reducers can sequester selenium (Klonowska et al. 2005; Pearce et al. 2009), and precipitate it as selenides, consistent with their occurrence instead of sulfides. Selenium may be co-precipitated with Fe (III) oxides (e.g., Howard 1977; Cabral et al. 2012), so on microbial reduction of the oxides both iron and selenium are liberated and the selenium is readily available for concentration and reprecipitation. Although selenium forms selenide anions, it behaves like a metal and is reduced by Fe (III) reducing bacteria along with other metals. Metalliferous cores in spheroids commonly show zonation of metals, in which selenides are concentrated towards the centre. The example in Fig. 3 shows zones rich in selenium, vanadium and uranium, all of which are precipitated by Fe (III) reducing bacteria (Holmes et al. 2002; Carpentier et al. 2003; Klonowska et al. 2005).

There is a consistency in composition of metallic cores for a locality or region, such as the silver-rich cores in Devon (Harrison 1975), and the uranium-rich cores in Nova Scotia (Dyck and McCorkell 1983). This indicates that pore fluids had homogenised at a local/regional scale, which requires some movement of the fluid through the rock prior to spheroid development. The reduction spheroids then developed within the context of the homogenised fluid. Metal precipitates in the spheroid cores may just involve a redistribution of metal within the volume of the spheroid, triggered by the reduction process. However, the metals precipitated do represent enrichment over a much wider body of rock, and thereby individual spheroid chemistries can be a pathfinder to regional metal anomalies.

## Potential Biosignatures on Earth and Mars

Given that the consequences of Fe (III) reduction have a visual expression, they are a potential biosignature during exploration of the terrestrial geological record. Fe (III) reducers are likely to be among the earliest widespread microbial inhabitants of Earth (Vargas et al. 1998; Kashefi and Lovley 2000). Reduction spheroids have a record back to the Mesoproterozoic (Tanton 1948; Dawes 1997; Spinks et al. 2010), so are a valuable record of microbial activity at a time when evidence for life is limited. They also show that the deep subsurface has been a habitat since early in the history of life. This early occurrence is constrained by the evolution of red beds due to increasing atmospheric oxygenation at the end of the Archean (Eriksson and Cheney 1992). Fe (III) reducing bacteria are also a potential life form (Liu et al. 1997), and the mineralogical expressions of bacterial Fe (III) reduction and Fe (II) oxidation are proposed as potential biosignatures, on other planets including Mars (Holden and Feinberg 2005; Weber et al. 2012). There is debate about the possible energy available from Fe (III) reduction on Mars (Nixon et al. 2012, 2013), but the abundance of iron in Martian soils makes it one of the most valuable prospects for life there. Entrapment of the microbes themselves as fossils is possible (Leveille and Lui 2009; Williams and Sumner 2013), but a more realistic target during the exploration of Mars would be the colour contrasts reflecting selective reduction or oxidation. It is distinctly possible that subsurface life on other rocky planets may be predominantly located in the subsurface, where there is protection from damaging irradiation and there is not the requirement of liquid surface water (McMahon et al. 2013). If that is so, then indicators of possible subsurface life such as reduction features may be a valuable target for exploration.

The oxidation of iron on the Martian surface is a conspicuous feature, and has been directly encountered by MER Opportunity at Meridiani Planum (Hurowitz et al. 2010) and MSL Curiosity (Blake et al. 2013) at Gale Crater, and more widely at Gale Crater by CRISM (Fraeman et al. 2013). Modelling of the evolution of sedimentary rocks at Gale Crater predicts iron oxide precipitation from the reaction of brines with country rock detritus (Bridges et al. 2013). The Gale Crater sedimentary rocks also include near-pure sulfates. Metalliferous terrestrial red beds also commonly contain sulfate and iron oxide associations, and similarly are believed to evolve through the reaction of brines with sedimentary detritus (Holmes et al. 1983). There are therefore important parallels between what is observed at Gale Crater and the settings where metalliferous biosignatures occur on Earth.

## Remote Analysis

Reduction features and associated metalliferous concentrations could be analysed by several techniques during remote exploration, including on Mars. The variations in colour related to the different mineralogy of iron in reduced and oxidised states mean that the features can be detected optically. Confirmation of the contrast in iron mineralogy can be achieved using instruments adapted for Martian missions, including Mössbauer spectroscopy (Dyar and Schaefer 2004), Raman spectroscopy (Pérez and Martinez-Frias 2006) and X-ray diffraction (Bish et al. 2013). The potential for Mössbauer measurements to record microbially-induced changes in iron oxidation state was emphasized by Schröder et al. (2006), using terrestrial deep sea samples, but is equally applicable to the detection of life in subsurface environments as proposed here.

### The Detection of Reduction Spheroids by Raman Spectroscopy

Raman spectroscopy has a wavelength range which covers most vibrational modes including metal oxides, carbonates, silicates and sulphates (i.e., most rock-forming minerals), therefore amongst its other many attributes, it can be used for petrographic analysis (Haskin et al. 1997; Wang et al. 1998). As previously discussed, the main factor controlling the colour contrast between the reduced halo of a reduction spot and the surrounding red rock, is the iron oxide coating on grain surfaces. Therefore a suitable target would be to analyse quartz grains across the redox boundary of a reduction spot. This can be achieved using Raman spectroscopy as it is sensitive to both quartz and hematite (De Faria et al. 1997; Ling et al. 2009). This could be applied to future landers which have spatial mapping capabilities, such as the NASA 2020 Raman instrument, SHERLOC (Beegle et al. 2015).

### Instrument Configuration

Raman spectra were obtained using a Renishaw InVia H36031 confocal Raman microscope operating at a wavelength of 514.5 nm green monochromatic laser light. A 50 × objective lens was used giving a laser “footprint” of 1–3 μ based on Beyssac et al. (2002). 10 s exposure time and 1 accumulation were used for each spectrum, giving a good signal to noise ratio with an extended spectral range of 100–2000 cm^−1^. A point analysis method was used, which transected a reduction spot from oxidised to reduced, back to oxidised areas. Representative spectra (3 from each oxidised and 4 from the reduced halo) were processed using a smooth, baseline subtraction and peak fit functions. This deconvolution process was repeated 3 times for each spectrum to account for human errors associated with manual baseline subtraction.

### Sample Collection & Preparation

A Permian oxidised siltstone from Budleigh Salterton, Devon, England was used with minimal sample preparation to simulate the ability of a rover during a remote mission. Therefore the sample was not cut or polished, and only reduction spots on natural bedding surfaces were analysed. The centimeter-scale size of the spots was appropriate for detection by a rover.

### Results and Interpretations

**Fig. 4:**
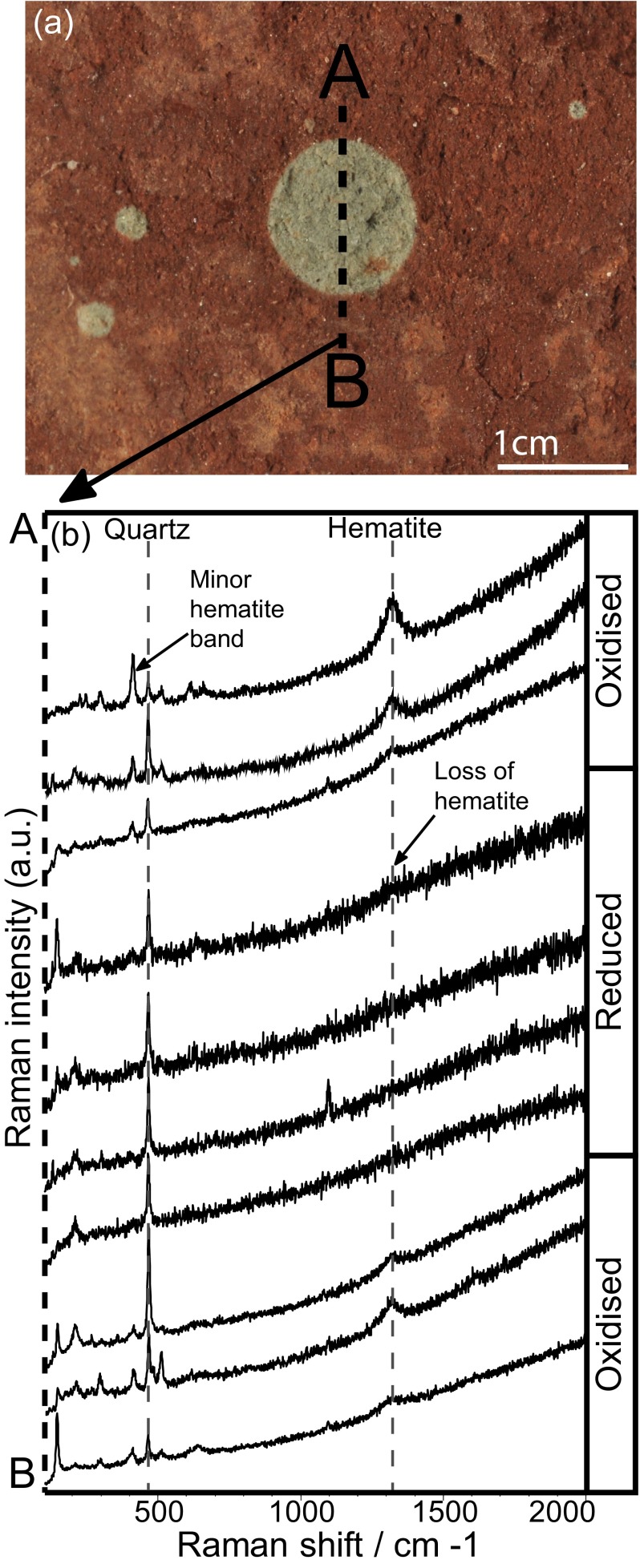
Raman spectroscopic transect of a reduction spheroid. **a** mm-scale Permian reduction spot from Budleigh Salterton, Devon with transect line A–B. **b** Representative spectra acquired by point analysis by Raman spectroscopy across transect line A–B. The *x*-*axis* is Raman shift, in reciprocal centimeters (cm-1) and the *y*-*axis* is Raman intensity, in arbitrary units (a.u.). Highlighted by the *dashed line* is the main spectral band for quartz and hematite. Spectra from the reduced areas show a weak or no hematite bands

Figure 4a shows a reduction spot from an oxidised siltstone from Budleigh Salterton with transect line A-B. Representative Raman spectra acquired across transect line A-B are displayed in Fig. 4b.

The main spectral bands observed are 198, 412, 462 and 1320 cm^−1^. Bands 198 and 462 cm^−1^ can be attributed to the quartz matrix. Bands at ~412 and 1320 cm^−1^ can be attributed to the hematite matrix. Therefore spectra from oxidised areas show a combination of bands attributed to hematite and quartz, as expected. Spectra which were acquired more proximal to the redox boundary display a reduction in band intensity for bands 412 and 1320 cm^−1^ (hematite), and an increase in band intensity of 462 cm^−1^ (quartz), which indicate that there is a reduction in hematite abundance. Spectra from within the reduction halo display major reduction in hematite band intensities, and a further increase in 462 cm^−1^ intensity, suggesting that the majority of the hematite has been removed by microbial action. Spectra acquired from the second oxidised portion show spectra similar to the first oxidised area and have a combination of spectra bands attributed to both hematite and quartz. This reduction feature is therefore adequately represented by the gradational disappearance and reappearance of the hematite bands observed in this Raman transect.

## Conclusion

Reduction spheroids are the most consistently encountered morphology of reduced sediment. Their formation is debated; however a microbial origin is the most likely. The spheroidal morphology indicates that they formed at depth, after compaction, which reflects the interaction of metals and microbes in the deep subsurface. Reduction spheroids can therefore be used as signature of life in the terrestrial geological record, but also can be applied to life on other planets. If life on other planets is predominantly subsurface, the reduction features may be a suitable biosignature to detect it. Due to the abundance of iron on Mars, it is a suitable target for putative microbial life which utilizes iron as an energy source. Detecting signatures of microbial iron reduction, such as reduction spheroids on Mars can be achieved by many techniques, including Raman spectroscopy. Point analysis transecting a reduction spot shows a clear difference between oxidised and reduced areas. Oxidised areas show a combination of spectral bands associated with quartz and hematite. Reduced areas show a weak or no hematite signal. This shows that Raman spectroscopy is able to detect the mineralogical changes associated with microbial iron reduction. It is not direct life detection, but is a valuable strategy to help find life and further promotes the application of Raman spectroscopy for remote missions to Mars.
